# Delirium in the Pediatric Cardiac Intensive Care Unit and Use of a Language Other than English

**DOI:** 10.1007/s00246-025-04016-y

**Published:** 2025-09-16

**Authors:** Mary S. Pilarz, Cara M. Pritchett, Quinn Tentinger, Jiafeng Li, Adrian D. Zurca

**Affiliations:** 1 Division of Critical Care, Ann & Robert H. Lurie Children’s Hospital of Chicago, 225 E Chicago Avenue, Chicago, IL 60611 USA; 2 Feinberg School of Medicine, Northwestern University, Chicago, USA; 3 Division of Cardiology, Ann & Robert H. Lurie Children’s Hospital of Chicago, Chicago, USA; 4 Stanley Manne Children’s Research Institute, Chicago, USA

**Keywords:** Delirium, Intensive Care Units, Pediatric, Critical Care, Communication Barriers, Language, Healthcare Disparities

## Abstract

This study aimed to characterize the relationship between the use of a language other than English (LOE) and the prevalence of delirium in the pediatric cardiac intensive care unit (CICU), hypothesizing that patients from families who use an LOE would have a higher prevalence of delirium compared to those from English-speaking families. This was a retrospective cohort study at a quaternary-care, free-standing children’s hospital CICU. Children admitted to the CICU between January 1, 2017, and July 31, 2023 with documented delirium screening scores were included. The primary outcome was the presence of delirium, assessed using the Cornell Assessment of Pediatric Delirium (CAP-D) score, with scores ≥ 9 indicating delirium. Language was recorded in the electronic health record and categorized as English, Spanish, or language other than English or Spanish (LOES). Covariates included age, sex, length of stay, developmental delay, use of mechanical ventilation, use of physical restraints, receipt of vasoactive medications, and caregiver presence. Among 1865 patients with CAP-D scores, 25.6% (477 patients) experienced delirium. Neither Spanish-speaking [adjusted OR (aOR) 1.43, 95% CI 0.92–2.22] nor LOES patients (aOR 0.73, 95% CI 0.33–1.60) demonstrated higher odds of delirium. Established risk factors for delirium, including age, length of stay, and mechanical ventilation, were associated with increased delirium risk. Inconsistent caregiver presence at bedside was associated with higher odds of delirium (aOR 2.37, 95% CI 1.76–3.18). In this CICU cohort, there was no significant association between the use of an LOE and delirium in the CICU. There is a strong, independent relationship between inconsistent caregiver presence and delirium.

## Background

Use of a language other than English (LOE) is common in the United States [1]. There is a known relationship between language barriers and lower quality health care, which include higher costs of care, longer lengths of stay, and more frequent adverse events [2–5]. Language barriers may be particularly harmful in the intensive care unit (ICU), where families often face complex medical decisions [6]. In the ICU, there are increased lengths of stay and increased mortality for Latino and Spanish-speaking patients [7, 8]. Families who use an LOE are more dissatisfied with the communication from staff, compared to families who use English [9].

Among patients admitted to pediatric ICUs, delirium is one of the most common complications of critical illness, and it is linked to increased ICU mortality, length of stay, and post-ICU morbidity [10–13]. Strategies to reduce delirium include involving families in rounds and direct patient care, like encouraging parental comforting; however, these efforts may be underutilized for families who use an LOE due to inadequate communication [14, 15]. As a result, patients from families who use an LOE may be at increased risk of delirium while in the ICU. No study to date has investigated language use as a possible risk factor for delirium.

This study aimed to characterize the relationship between a family’s preferred language and delirium in the pediatric cardiac intensive care unit. We hypothesized that there would be an increased prevalence of delirium among the patients whose families used an LOE compared to the English-speaking population.

## Methods

### Study Design and Participants

This was a retrospective cohort study of patients admitted to the cardiac intensive care unit (CICU) at an urban, quaternary-care, free-standing children’s hospital from January 1, 2017 to July 31, 2023. The study included all patients admitted to the CICU who had delirium screening performed; patients who were missing delirium scoring were excluded. The CICU was specifically chosen as the site of the study due to more robust delirium screening practices than the non-cardiac ICU. The Ann and Robert H. Lurie Children’s Hospital of Chicago Institutional Review Board waived the need for approval (IRB 2023-6455) and informed consent.

### Study Variables

#### Language

A family’s preferred language was defined using data from the electronic health record (EHR). Language use was categorized as English, Spanish, and language other than English or Spanish (LOES). We analyzed Spanish-speaking patients and LOES separately because of the known differences in access to interpretation for Spanish compared to other languages and worse outcomes for the LOES population [10].

### Delirium Scoring

Patients were categorized as having delirium if they had at least one Cornell Assessment of Pediatric Delirium (CAP-D) score of 9 or greater during their CICU hospitalization [11]. Staff were trained on delirium scoring through handouts, live presentations, and pre-shift huddles. Competency in delirium assessment and documentation was evaluated using a standardized tool. Laminated reference materials were placed at each patient’s bedside.

### Covariates

We accounted for length of stay in the analysis, as well as known risk factors for delirium, including any diagnosis of developmental delay, need for mechanical ventilation, use of physical restraints, and receipt of medications linked to delirium (benzodiazepines, opiates, steroids, or vasoactive medication) [12]. The presence of a caregiver at bedside was also included, as measured by hourly nursing charting. If a caregiver was charted as present 80% of the time or more, they were categorized as present, based on prior literature about family and caregiver presence at the bedside [16].

### Statistical Analysis

Descriptive statistics were calculated for all encounters and included frequencies and percentages for categorical data and medians and ranges for continuous variables. In univariate analysis, the *χ*^2^ test was used for categorical variables and the Wilcoxon test for continuous variables, as the latter were not normally distributed. We used a mixed-effects logistic regression model to analyze the association between the delirium status and language with covariates determined a priori based on known risk factors for delirium. We used both fixed effects and random effects in the model because patients were included multiple times if they had multiple admissions. All statistical analysis was conducted using R software. *p* values ≤ 0.05 were considered statistically significant.

## Results

### Study Population

There were 2990 patient encounters and 1983 unique patients who were admitted to the CICU during the study period. Of that group, 1865 patient encounters (62.4%) had recorded CAP-D scores during the CICU admission. Patients from families who used an LOE were screened more often than patients from English-speaking families (251/369 [68%] vs. 1614/2621 [62%], *p* = 0.02). Of the patient encounters that received CAP-D scoring, 1614 (86.5%) preferred English, 186 (10.0%) used Spanish, and 65 (3.5%) used an LOES. Delirium was present in 477 (25.6%) of patient encounters that were screened (Table 1).

**Table 1:** Characteristics of encounters with and without delirium

Encounter Characteristics	Delirium present	Delirium absent	*p* value
Age in years, median (IQR)	*N* = 477	*N* = 1388	< 0.001*
	0.32 (0.01, 1.66)	1.51 (0.33, 10.12)	
Sex, *n* (%)			0.403
Male	271 (56.8)	756 (54.5)	
Female	206 (43.2)	632 (45.5)	
Insurance, *n* (%)			0.362
Private	198 (41.5)	622 (44.8)	
Public	265 (55.6)	737 (53.1)	
Government	4 (0.8)	12 (0.9)	
Other	10 (2.1)	17 (1.2)	
Language, *n* (%)			0.498
English	413 (86.6)	1201 (86.5)	
Spanish	51 (10.7)	135 (9.7)	
Other	13 (2.7)	52 (3.8)	
Race/ethnicity,* n* (%)			0.514
Asian	20 (4.2)	86 (6.2)	
Black	97 (20.3)	270 (19.5)	
Hispanic	145 (30.4)	394 (28.4)	
Multiracial	13 (2.7)	29 (2.1)	
Other	13 (2.7)	46 (3.3)	
White	189 (39.6)	563 (40.6)	
Length of stay in days, median (IQR)	22.5 (7.08, 83.54)	3.83 (0.79, 9.84)	< 0.001*
Received medications associated with delirium, *n* (%)	451 (94.5)	1091 (78.6)	< 0.001*
Developmental delay, *n* (%)	282 (59.1)	682 (49.1)	< 0.001*
Highest CAP-D score, median (IQR)	9 (9, 9)	4 (1, 6)	
Lowest CAP-D score, median (IQR)	0 (0, 10)	0 (0, 10)	
Total number of CAP-D scores documented, median (IQR)	35 (12, 99)	6 (3, 15)	< 0.001*
Ventilator days, median (IQR)	11 (1, 45)	0 (0, 1)	< 0.001*
Caregiver present, *n* (%)			< 0.001*
Yes	137 (28.7)	777 (56.0)	
No	340 (71.2)	611 (44.0%)	

*p value ≤ 0.05

### Delirium and Language

In univariate analysis, neither patients from Spanish-speaking families (OR 1.13, 95% CI 0.78–1.65) nor patients from LOES families (OR 0.74, 95% CI 0.38–1.46) had higher odds of delirium compared to children from families who used English (Table 1). Patients who screened positive for delirium were younger and had a longer length of stay. These patients also had higher rates of receiving medications associated with delirium, had higher rates of developmental delay, had more ventilator days, and were less likely to have a caregiver present at bedside.

After adjusting for age, sex, length of stay, medications, developmental delay, and ventilator days, neither speaking Spanish (aOR 1.43, 95% CI 0.92–2.22) nor speaking other languages (aOR 0.73, 95% CI 0.33–1.60) were significantly associated with the presence of delirium. Age, length of stay, receipt of vasoactives and/or steroids, developmental delay, and days of mechanical ventilation were all associated with increased odds of delirium (Table 2). Inconsistent caregiver presence at bedside was associated with higher odds of delirium (aOR 2.37, 95% CI 1.76–3.18).

**Table 2:** Multivariable logistic regression for the presence of delirium

Encounter characteristics	Odds ratio	95% CI
Language		
English	Ref.	Ref.
Spanish	1.429	(0.919, 2.220)
Other	0.728	(0.331, 1.600)
Age^a^	0.929	(0.907, 0.952)*
Sex		
Male	Ref.	Ref.
Female	0.936	(0.713, 1.229)
Length of stay	1.007	(1.004, 1.011)*
Receipt of deliriogenic medications	3.111	(1.964, 4.926)*
Developmental delay	1.359	(1.034, 1.787)*
Ventilator days	1.031	(1.022, 1.040)*
Caregiver at bedside		
Yes	Ref.	Ref.
No	2.366	(1.76, 3.18)*

*p value of ≤ 0.05

^a^For each additional year of age, the odds ratio of delirium presence decreases by a factor of 0.929, indicating younger age is associated with slightly higher odds of delirium

## Discussion

In this single-site, retrospective study, there was no association between familial use of an LOE and the presence of delirium in the CICU. This finding was contrary to our initial hypothesis that familial use of an LOE would be associated with delirium. Delirium in the CICU is multifactorial, influenced by medical, environmental, and psychosocial factors that may outweigh the impact of language barriers alone [14].

Screening practices may have influenced the results. In our study population, 25.6% of patients screened positive for delirium. However, 37.6% of patients did not receive any CAP-D scoring, and patients from families who used English were less likely to be screened. This rate of missed screening is higher than other published studies, which was 26% in a recent multicenter study [17]. Previous studies of delirium in the CICU have found the prevalence of delirium to be as high as 67% with a screening rate of 98% [18]. Inadequate screening may have limited our ability to detect a disparity in delirium by language preference. Additionally, there were quality improvement projects during the study period; screening rates increased between 2019 and 2020. Our cardiac unit has an “acuity-adaptable” model, meaning children stay in the CICU throughout their admission; it may be that children who were less critically ill did not receive consistent delirium screenings.

This study demonstrates an association between inconsistent caregiver presence at bedside and higher odds of delirium in a pediatric ICU, which has previously been described in one other pediatric single-center study [19]. This finding bolsters the recommendations that caregiver presence be facilitated in pediatric ICU to prevent delirium [14]. Familial presence at bedside has been associated with sociodemographic and illness factors in prior work, and working toward equity in family presence is critical to mitigating disparities in delirium in the ICU [16].

Our study does have several important limitations. Most importantly, it was single site and retrospective, which limits its generalizability. It is also important to note that our study was conducted in the cardiac ICU as opposed to the general pediatric ICU because at the study institution, there is more consistent delirium screening in the cardiac ICU. However, only 62.4% of CICU patients in our study period received a CAP-D score, so patients with delirium may not have been scored.

Language proficiency exists on a spectrum, and dichotomizing families into English or LOE use may misclassify those with varying proficiency. Our exposure also only reflects family language preference, not patient language preference. Although the cohort included children 0–18 years, most were preverbal, so the analysis primarily captures the effect of family–clinician communication. Adult and geriatric studies have shown that language discordance between patients and clinicians increases delirium risk and restraint use, likely by exacerbating confusion and disorientation [20]. While our study extends this inquiry to a pediatric ICU population, conclusions should be tempered given the age distribution of the cohort; future research is needed in verbal children to assess direct patient–clinician language effects.

## Conclusion

Although this study did not demonstrate an association between the use of an LOE and delirium, further work is required to investigate this patient population that is vulnerable to disparities. Our findings highlight caregiver presence as a modifiable risk factor for delirium and underscore the need for equitable family engagement in the ICU.

## Data Availability

The data that support the findings of this study are not openly available due to reasons of sensitivity and are available from the corresponding author upon reasonable request. Data are located in controlled access data storage at Ann & Robert H. Lurie Children's Hospital of Chicago.

## Abbreviations

**LOE**: Languages other than English
IRB: Institutional Review Board (IRB)
EHR: Electronic health record
